# Gender-specific relationship between thigh muscle and fat mass and brain amyloid-β positivity

**DOI:** 10.1186/s13195-022-01086-5

**Published:** 2022-10-04

**Authors:** Sung Hoon Kang, Kyung Hyun Lee, Yoosoo Chang, Yeong Sim Choe, Jun Pyo Kim, Hyemin Jang, Hee Young Shin, Hee Jin Kim, Seong-Beom Koh, Duk L. Na, Sang Won Seo, Mira Kang

**Affiliations:** 1 Department of Neurology, Samsung Medical Center, Sungkyunkwan University School of Medicine, Seoul, South Korea; 2 Department of Neurology, Korea University Guro Hospital, Korea University College of Medicine, Seoul, South Korea; 3 Department of Digital Health, SAIHST, Sungkyunkwan University, Seoul, South Korea; 4 Center for Cohort Studies, Total Healthcare Center, Kangbuk Samsung Hospital, Sungkyunkwan University School of Medicine, Seoul, South Korea; 5 Department of Health Sciences and Technology, SAIHST, Sungkyunkwan University, Seoul, South Korea; 6 Center for Health Promotion, Samsung Medical Center, Sungkyunkwan University School of Medicine, Seoul, South Korea; 7 Alzheimer’s Disease Convergence Research Center, Samsung Medical Center, Seoul, South Korea; 8 Department of Intelligent Precision Healthcare Convergence, Sungkyunkwan University, Suwon, South Korea; 9 Digital Innovation Center, Samsung Medical Center, Sungkyunkwan University School of Medicine, Seoul, South Korea

**Keywords:** Amyloid-β (Aβ), Muscle, Fat, Thigh, Gender

## Abstract

**Background:**

The relationship of specific body composition in the thighs and brain amyloid-beta (Aβ) deposition remained unclear, although there were growing evidence that higher muscle and fat mass in thighs had a protective effect against cardiometabolic syndromes. To determine whether muscle mass and fat mass in the thighs affected amyloid-beta (Aβ) positivity differently in relation to gender, we investigated the association of muscle mass and fat mass with Aβ positivity using positron emission tomography (PET) in individuals without dementia.

**Methods:**

We recruited 240 participants (134 [55.8%] males, 106 [44.2%] females) without dementia ≥45 years of age who underwent Aβ PET, bioelectrical impedance analysis (BIA) and dual-energy X-ray absorptiometry (DEXA) scans of the hip in the health promotion center at Samsung Medical Center in Seoul, Korea. Lower extremity skeletal muscle mass index (LASMI) was measured using BIA, and gluteofemoral fat percentage (GFFP) was estimated using DEXA scans of the hip. We investigated the associations of LASMI and GFFP with Aβ positivity using logistic regression analyses after controlling for age, *APOE4* genotype, and cognitive stage.

**Results:**

Higher muscle mass in the thighs, measured as LASMI (odds ratio [OR]=0.27, 95% confidence interval [CI] 0.08 to 0.84, *p*=0.031) was associated with a lesser risk of Aβ positivity in only females. Higher fat mass in the thighs, measured as GFFP (OR=0.84, 95% CI 0.73 to 0.95, *p*=0.008) was associated with a lesser risk of Aβ positivity in only males. However, the association between LAMSI (*p for interaction*= 0.810), GFFP (*p for interaction*= 0.075) and Aβ positivity did not significantly differ by gender. Furthermore, LAMSI only negatively correlated with centiloid (CL) values in females (*r*=−0.205, *p*=0.037), and GFFP only negatively correlated with CL values only in males (*r*=−0.253, *p*=0.004).

**Conclusions:**

Our findings highlight the importance of recognizing that gender differences exist with respect to the specific body composition to potentially protect against Aβ deposition. Therefore, our results may help in designing gender-specific strategies for controlling body composition to prevent Aβ deposition.

**Supplementary Information:**

The online version contains supplementary material available at 10.1186/s13195-022-01086-5.

## Background

Deposition of amyloid-beta (Aβ) is the most prominent pathological change in Alzheimer’s disease (AD). According to many studies on in-vivo Aβ biomarkers, brain Aβ deposition precedes brain atrophy and cognitive impairment. Cognitively normal participants with Aβ biomarkers are reported to be more likely to develop mild cognitive impairment (MCI) or dementia than those without Aβ biomarkers [1–3]. Thus, Aβ positivity is an important predictor of AD dementia prognosis.

Growing body of evidence shows that weight loss is associated with a higher risk of AD [4, 5]. Weight loss is also reported to be the early sign of AD [6]. Furthermore, there were several studies investigating the associations of BMI or weight loss with Aβ deposition [7, 8]. However, since BMI reflects nonspecific measures of body composition, previous studies using BMI were not able to investigate which body components (ie, muscle mass and fat mass) might be associated with a higher risk of Aβ deposition.

Previous studies have suggested that thigh circumference may be an important determinant of cardiometabolic syndrome [9] and mortality [10]. Both increased fat and muscle mass in the thighs, which reflect for a large composition of human body [11, 12], have protective effects against cardiometabolic syndrome [13, 14]. Specifically, higher fat mass in the thighs was found to be associated with a better metabolic marker status including higher adiponectin levels, lower insulin resistance, lower cholesterol levels, and lower C-reactive protein levels [15–18]. Higher thigh fat mass also prevents cardiometabolic syndrome, including hypertension, diabetes, and hyperlipidemia [19, 20]. In contrast, progressive loss of muscle mass in the thighs with aging, which is known as sarcopenia [21], is associated with a greater risk of cardiovascular disease [22, 23]. In addition, there is increasing evidence that lower muscle mass is related to metabolic impairment and poor health outcomes, including increased morbidity and mortality rates [24, 25]. Considering that cardiometabolic syndromes are closely associated with the development of AD, it would be reasonable to expect that decreased fat and muscle mass of the thighs might be associated with increased risk of Aβ deposition in individuals without dementia. Furthermore, previous studies have revealed differences in body composition between males and females. In fact, it was found that fat mass was lower and muscle mass was higher in males than in females [14]. Therefore, it is possible that Aβ deposition in males might be vulnerable to decreased fat mass, while Aβ deposition in females might be vulnerable to decreased muscle mass.

The objective of our study was to investigate the association of muscle mass in the thighs, as measured using bioelectrical impedance analysis (BIA), and fat mass in the thighs, as measured using dual-energy X-ray absorptiometry (DXA) scans of the lower body, with Aβ positivity using positron emission tomography (PET) in individuals without dementia. Specifically, we determined whether fat and muscle mass in the thighs affected Aβ positivity differently between males and females.

## Methods

### Study Participants

We enrolled 240 participants without dementia ≥45 years of age who underwent a full health screening examination, including BIA and DXA scans of the hip in the health promotion center at Samsung Medical Center (Seoul, Korea) between August 2015 and October 2020. All patients also underwent standardized neuropsychological test battery using the Seoul Neuropsychological Screening Battery 2nd edition (SNSB-II) [26, 27], blood tests, brain magnetic resonance imaging (MRI), and Aβ PET. Participants without dementia were participants with normal cognition (NC) and those with MCI. All participants with NC met the following criteria: (1) no medical history that was likely to affect cognitive function based on Christensen's health screening criteria [28]; (2) no objective cognitive impairment in any cognitive domain on a comprehensive neuropsychological test battery (above at least -1.0 SD of age-adjusted norms on any cognitive test); and (3) independence in activities of daily living. All patients with MCI met the criteria for MCI with the following modifications [29, 30]: (1) subjective cognitive complaints by the participants or caregivers; (2) objective memory impairment below -1·0 SD on verbal or visual memory tests, (3) no significant impairment in activities of daily living, and (4) non-demented status.

We excluded participants with significant whiter matter hyperintensities (cap or band >10 mm and longest diameter of deep white matter lesion > 25 mm), structural lesions including cerebral infarction, intracranial hemorrhage, brain tumors, and hydrocephalus on MRI, and abnormal laboratory results on complete blood count, electrolyte, vitamin B12 and folate levels, syphilis serology, and liver/kidney/thyroid function tests.

All participants underwent BIA and DXA scans of the hip on the same day, and these measurements were performed within three years before or after the Aβ PET. The median time interval between the measures was 4 months (interquartile range, 2–7 months). Two hundreds and twelve (88.3%) out of 240 participants had less than 12-month interval between BIA and DXA scans and Aβ PET.

The institutional review board of the Samsung Medical Center approved this study. Written informed consent was obtained from all participants.

### Aβ PET acquisition

All participants underwent Aβ PET (^18^F-florbetaben PET and ^18^F-flutemetamol PET scans using a Discovery STe PET/CT scanner (GE Medical Systems, Milwaukee, WI, USA). For ^18^F-florbetaben PET or ^18^F-flutemetamol PET, a 20-minute emission PET scan in dynamic mode (consisting of 4 × 5 min frames) was performed 90 min after an injection of a mean dose of 311.5 MBq ^18^F-florbetaben or 197.7 MBq ^18^F-flutemetamol, respectively. Three-dimensional PET images were reconstructed in a 128 × 128 × 48 matrix with 2 mm × 2 mm × 3·27 mm voxel size using the ordered-subsets expectation maximization algorithm (^18^F-florbetaben, iteration = 4 and subset = 20; ^18^F-flutemetamol, iteration = 4 and subset = 20).

### Aβ PET quantification using centiloid values

We used a centiloid (CL) method previously developed by our group [31] to standardize the quantification of Aβ PET images obtained using different ligands. The CL method for FBB and FMM PET enables the transformation of the standardized uptake value ratio (SUVR) of FBB and FMM PETs to CL values directly without conversion to the ^11^C-labeled Pittsburgh compound SUVR.

There are three steps to obtain CL values [31]: (1) pre-processing of PET images, (2) determination of CL global cortical target volume of interest (CTX VOI), and (3) conversion of SUVR to CL values. First, to pre-process the Aβ PET images, PET images were co-registered to each participant’s MR image and then normalized to a T1-weighted MNI-152 template using the SPM8 unified segmentation method. We used T1-weighted MR image correction with the N3 algorithm only for intensity nonuniformities, without applying corrections to the PET images for brain atrophy or partial volume effects. Second, we used the FBB-FMM CTX VOI defined as areas of AD-specific brain Aβ deposition in our previous study [31]. Briefly, to exclude areas of aging-related brain Aβ deposition, the FBB-FMM CTX VOI was generated by comparing SUVR parametric images (with the whole cerebellum as a reference area) between 20 typical patients with Alzheimer’s disease-related cognitive impairment (AD-CTX) and 16 healthy elderly participants (EH-CTX) who underwent both FBB and FMM PET scans. To generate the FBB-FMM CTX VOI, the average EH-CTX image was subtracted from the average AD-CTX image. We then defined the FBB-FMM CTX VOI as areas of AD-related brain Aβ accumulation common to both FBB and FMM PET. Finally, the SUVR values of the FBB-FMM CTX VOI were converted to CL units using the CL conversion equation. The CL equation was derived from the FBB-FMM CTX VOI separately for FBB and FMM PET and applied to the FBB and FMM SUVR.

To determine the participants’ CL cut-off-based Aβ positivity, we applied the optimal cut-off value derived using a k-means cluster analysis in 527 independent samples of participants with normal cognition. The cut-off value was set at 27.08, representing the 95th percentile of the lower cluster [32], and the whole cerebellum was used as a reference region.

### Thigh muscle mass measurement

To obtain body mass index (BMI) and appendicular skeletal muscle mass (ASM) of the bilateral lower limbs, all participants underwent BIA using a multifrequency BIA device (InBody 720; InBody Co., Ltd., Seoul, Korea) according to the manufacturer’s guidelines [33]. The appendicular skeletal muscle mass index in the lower extremity (LASMI) was calculated by dividing the sum of the ASM in the bilateral lower limbs by the square of the height (ASM in bilateral lower extremity/[height]^2^). According to previous results that LASMI was highly correlated with thigh muscle volume in MRI scans of thigh [34], we considered LASMI as a proxy marker of muscle mass in the thigh.

### Thigh fat mass measurement

All participants underwent DXA scans using Lunar Prodigy Advance (GE Healthcare, Madison, WI, USA) according to the manufacturer’s guidelines. DXA scans are widely used diagnostic tools for osteoporosis and are recommended in the elderly. The left side was scanned routinely, and for participants with left hip fracture or device, the right side was scanned. The boundaries of the regions of interest (ROIs) for determining regional body composition were automatically defined using the software provided by the manufacturer (software version: enCORE version 18.0). We selected the gluteofemoral region as the ROI, and the fat percentage of the soft tissue in the gluteofemoral region included in the hip scans was extracted from the scanner databases. We considered the gluteofemoral fat percentage (GFFP) as a proxy marker of fat mass in the thigh.

### Standardized neuropsychological test battery

All participants underwent the SNSB-II [26, 27], which includes standardized and validated tests of various cognitive functions. The SNSB-II evaluates lots of cognitive factors including verbal and visual memory, visuo-constructive function, language, praxis, components of Gerstmann syndrome (acalculia, agraphia, right/left disorientation, finger agnosia), and frontal/executive functions. We chose to use six cognitive measures, which were representative and important neuropsychological tests to evaluate the cognitive function in five cognitive domains as follows: (1) Memory: the Seoul Verbal Learning Test SVLT delayed recall and Rey-Osterrieth Complex Figure Test (RCFT) delayed recall; (2) Language: Korean version of the Boston Naming Test; (3) Visuospatial function: RCFT copying Test; (4) Frontal executive function: the Stroop Test color reading; and (5) Attention: Digit Span Test backward.

### Statistical analyses

All statistical analyses were performed separately in males and females. We used independent t-tests and chi-squared tests to compare the demographic and clinical characteristics of Aβ-positive (Aβ+) and Aβ-negative (Aβ−) groups. To explore the association between BMI and Aβ positivity, we performed a logistic regression analysis with BMI as predictor after controlling for age, *APOE4* genotype, and cognitive stage (NC and MCI). To check the correlation among BMI, LASMI, and GFFP, we used Pearson’s correlation with BMI as a dependent variable and LASMI or GFFP as an independent variable. To investigate the association between body composition (muscle and fat mass) in the thighs and Aβ positivity, we performed a logistic regression analysis with LASMI and GFFP together as predictors after controlling for age, *APOE4* genotype, and cognitive stage (NC and MCI). To further validate the relationship between Aβ burden and body composition (muscle and fat mass) in the thighs, we explored the relationship using quantified CL values rather than cut-off-based categorization. In this analysis, we used partial correlation with CL values as dependent variables and LASMI or GFFP as independent variables after controlling for age, *APOE4* genotype, and cognitive stage (NC and MCI). Finally, to evaluate the effect modification of gender and body composition in the thighs on Aβ positivity, we performed multivariable logistic regression analysis with LASMI, GFFP, and gender together as main effects and LASMI*gender and GFFP*gender as interaction effects after controlling for age and *APOE4* genotype in all participants (males and females).

All reported *p*-values were two-sided, and the significance level was set at 0.05. All analyses were performed using R version 4.3.0 (Institute for Statistics and Mathematics, Vienna, Austria; www.R-project.org).

## Results

### Clinical characteristics of participants

Among the 240 participants, there were 134 males with a mean age of 71.3±6.7 years and 106 females with a mean age of 69.9±8.1 years (Table 1). There were no differences between males and females in the frequency of Aβ+ (32.8% and 38.7%, *p*=0.421) (Table 1) and in the frequency of MCI stage (53.7% and 42.5%, *p*=0.108) (Additional file 1: Table S1). Males (13.7±4.0) had a significantly higher mean years of education than females (12.0±4.8, *p*=0.002). However, rates of hypertension, diabetes, and *APOE4* genotype were not different between males and females. Males (5.57±0.47) had a higher mean LASMI than females (4.64±0.44, *p*<0.001) and females (25.19±4.54) had a higher mean GFFP than males (18.72±4.05, *p*<0.001), although mean BMI was not different between gender (*p*=0.166).

**Table 1:** Demographic variables and body composition profiles of study participants

Variables	Males			Females		
	Total (*n* = 134)	Aβ (−) (*n* = 90)	Aβ (+) (*n* = 44)	Total (*n*= 106)	Aβ (−) (*n* = 65)	Aβ (+) (*n* = 41)
*Cognitive stage*						
NC	62 (46.3%)	49 (54.4%)^∗^	13 (29.5%)^∗^	61 (57.5%)	43 (66.2%)^∗^	18 (43.9%)^∗^
MCI	72 (53.7%)	41 (45.6%)^∗^	31 (70.5%)^∗^	45 (42.5%)	22 (33.8%)^∗^	23 (56.1%)^∗^
*Demographics*						
Age, years	71.3±6.7	70.5±7.2^∗^	72.8±5.3^∗^	69.9±8.1	68.9±8.3	71.5±7.5
Education, years	13.7±4.0	14.2±3.5	12.8±4.6	12.0±4.8	12.4±4.7	11.2±4.9
*APOE*, *e4* carrier	47 (35.1%)	19 (21.1%)^∗^	28 (63.6%)^∗^	33 (31.1%)	10 (15.4%)^∗^	23 (56.1%)^∗^
Hypertension	64 (47.8%)	46 (51.1%)	18 (40.9%)	49 (46.2%)	29 (44.6%)	20 (48.8%)
Diabetes	32 (23.9%)	23 (25.6%)	9 (20.5%)	17 (16.0%)	11 (16.9%)	6 (14.6%)
*Body composition*						
BMI, kg/m^2^	24.0±2.6	24.56±2.57^∗^	22.85±2.41^∗^	23.5±2.9	24.01±2.91^∗^	22.69±2.84^∗^
LASMI, kg/m^2^	5.57±0.47	5.62±0.50	5.48±0.39	4.64±0.44	4.72±0.42^∗^	4.50±0.44^∗^
GFFP, %	18.72±4.05	19.48±4.02^∗^	17.15±3.66^∗^	25.19±4.54	25.28±4.08	25.05±5.24
*Aβ deposition*						
Centiloid	27.0±42.1	1.3±6.7^∗^	79.6±34.2^∗^	27.1±37.1	2.2±6.3^∗^	66.4±30.9^∗^
*Cognitive tests*						
SVLT recall	4.4±2.9	5.2±2.9^∗^	2.9±2.3^∗^	5.2±3.4	6.1±3.1^∗^	3.5±3.2^∗^
RCFT recall	13.2±6.9	15.0±7.0^∗^	9.7±5.1^∗^	11.0±6.9	13.0±6.7^∗^	7.8±6.1^∗^
K-BNT	46.7±7.8	47.7±7.3^∗^	44.7±8.5^∗^	43.3±9.7	45.1±8.6^∗^	40.3±10.8^∗^
RCFT copying	32.2±4.5	32.9±3.1^∗^	30.7±6.2^∗^	30.4±7.2	31.7±5.7^∗^	28.3±8.8^∗^
DSB	3.9±1.0	3.9±1.0	3.9±1.0	3.9±1.4	4.1±1.5	3.7±1.3
Stroop CR	73.0±24.9	76.1±23.0^∗^	66.8±27.6^∗^	80.5±28.5	88.0±25.7^∗^	68.0±28.7^∗^

Values are presented as mean ± standard deviation

*Abbreviations*: *Aβ (−)* amyloid negative, *Aβ (+)* amyloid positive, *n* number of patients whose data were available for analysis, *BMI* body mass index, *CR* color reading, *DSB* Digit Span Test backward, *LASMI* lower extremity appendicular skeletal muscle mass index, *GFFP* gluteofemoral fat percentage, *K*-*BNT* Korean version of the Boston Naming Test, *RCFT* Rey-Osterrieth Complex Figure Test, *SVLT *Seoul Verbal Learning Test delayed recall

^∗^Significant difference at *p*<0.05 between Aβ (−) and Aβ (+) in the same gender

### Effect of BMI on Aβ uptakes

Among males, the Aβ+ group had (22.85±2.41) lower BMI than Aβ- (24.56±2.57, *p*<0.001). Among females, the Aβ+ group also had (22.69±2.84) lower BMI than Aβ- (24.01±2.91, *p*=0.024). BMI was also associated with Aβ positivity in both males (OR=0.76, 95% CI 0.61 to 0.92) and females (OR=0.82, 95% CI 0.68 to 0.96).

Regarding the relationship of BMI with LASMI and GFFP, among males, BMI was positively correlated with LASMI (*r*=0.581, *p*<0.001) and GFFP (*r*=0.422, *p*<0.001). Among females, BMI was also correlated with LASMI (*r*=0.533, *p*<0.001) and GFFP (*r*=0.349, *p*=0.001)

### Effects of body composition on Aβ uptakes

**Fig. 1:**
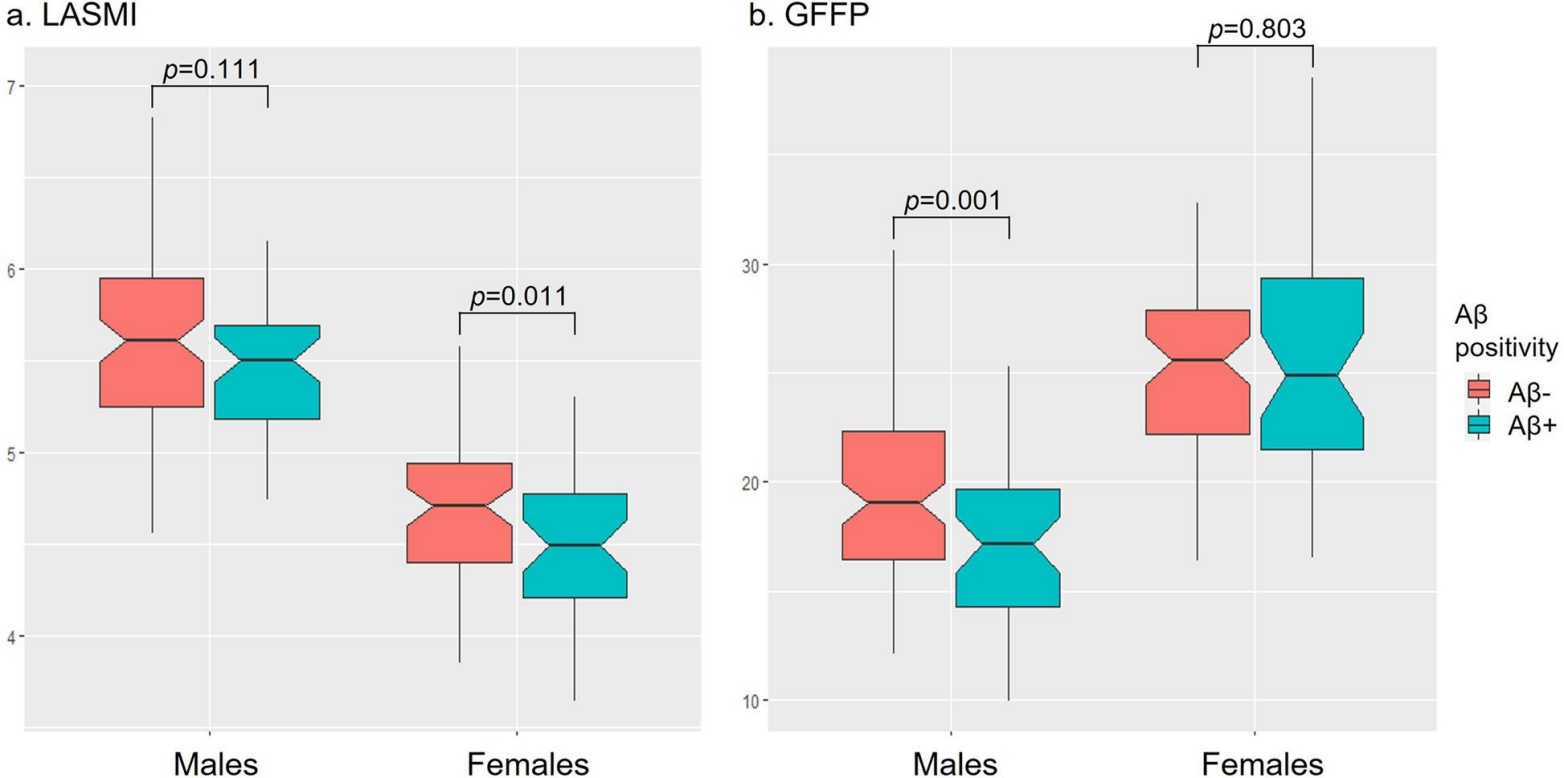
Gender differences in muscle and fat mass between Aβ positivity. **a** Values depicted in the box plot represent LASMI on the *Y*-axis. **b** Values depicted in the box plot represent GFFP on the *Y*-axis. Abbreviations: Aβ, amyloid; OR, odds ratios; LASMI, lower extremity muscle mass; GFFP, gluteofemoral fat percentage

As illustrated in Fig. 1, among males, Aβ+ group (17.15±3.66) had lower GFFP than Aβ- (19.48±4.02, *p*=0.001), while mean LASMI was not different between Aβ+ and Aβ- group (*p*=0.111). Among females, Aβ+ group (4.72±0.42) had lower LASMI than Aβ- (4.50±0.44, *p*=0.011), while mean GFFP was not different between Aβ+ and Aβ- group (*p*=0.803).

Among males, GFFP (OR=0.84, 95% CI 0.73 to 0.95) was independently associated with Aβ positivity, while LASMI (OR=0.49, 95% CI 0.16 to 1.40) was not associated with Aβ positivity (Table 2). In contrast, among females, a higher LAMSI (OR=0.27, 95% CI 0.08 to 0.84) was independently associated with lesser Aβ positivity, while GFFP (OR=0.99, 95% CI 0.89 to 1.10) was not associated with Aβ positivity (Table 2). There was no interaction effect of gender with LAMSI (gender*LASMI, *p*=0.875) and GFFP (gender*GFFP, *p*=0.092) on Aβ positivity in all participants (Table 2).

**Table 2:** Aβ positivity based on body composition

		Males		Females		
		OR (95%CI)^a^	*p*	OR (95%CI)^a^	*p*	*p for interaction by gender*^b^
Muscle	LASMI	0.49 (0.16–1.40)	0.192	0.27 (0.08–0.83)	0.031	0.810
Fat	GFFP	0.84 (0.73–0.95)	0.008	0.98 (0.89–1.10)	0.870	0.075

*Abbreviations*: *Aβ* amyloid, *OR* odds ratio, *BMI* body mass index, *LASMI* lower extremity muscle mass, *GFFP* gluteofemoral fat percentage, *NC* normal cognition, *MCI* mild cognitive impairment

^a^Adjusted OR for Aβ positivity was obtained by the logistic regression analysis with LASMI and GFFP together as predictors after controlling for age, APOE4 genotype, and cognitive stage (NC and MCI)

^b^*p* for interaction was estimated by the logistic regression analysis including LASMI, GFFP and gender together as main effects and gender*LASMI and gender*GFFP as interaction effects after controlling for age, APOE4 genotype, and cognitive stage (NC and MCI)

**Fig. 2:**
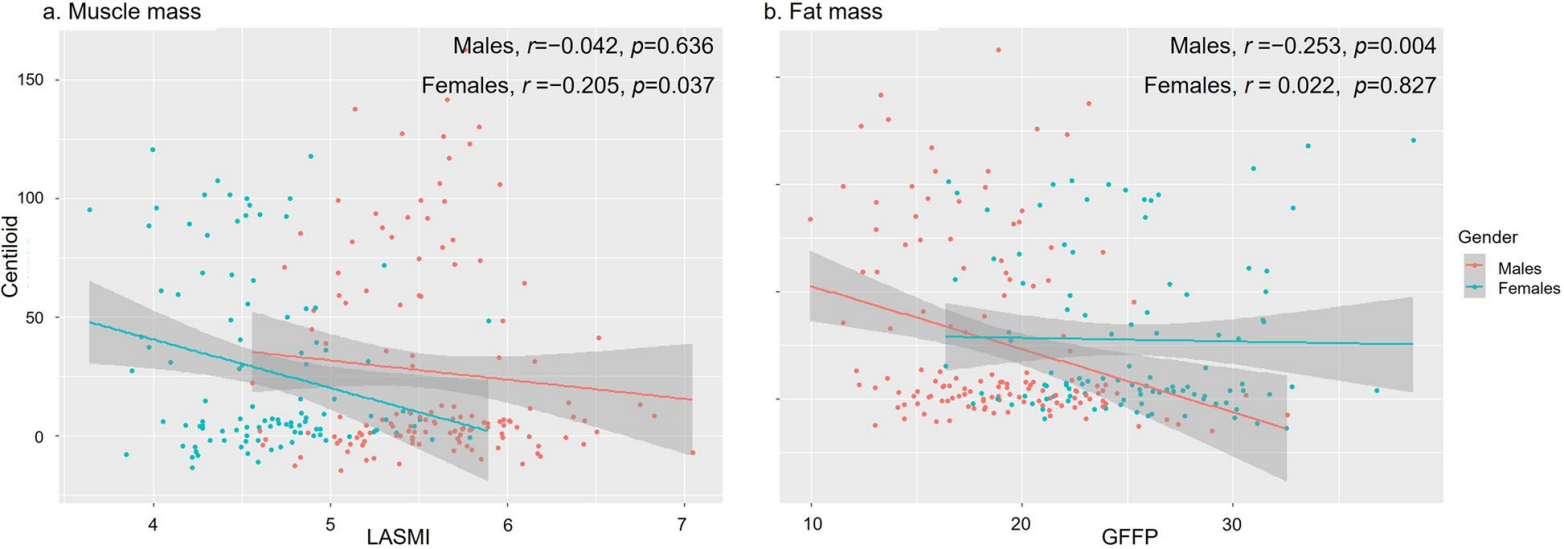
Correlations between Aβ PET CL values and muscle or fat mass in lower extremities in men and women. **a** Values depicted in the scatter plot represent LASMI on the *X*-axis and Aβ PET CL values on the *Y*-axis. **b** Values depicted in the scatter plot represent GFFP on the *X*-axis and Aβ PET CL values on the *Y*-axis. Abbreviations: Aβ, amyloid; CL, centiloid; LASMI, lower extremity appendicular skeletal muscle mass index. GFFP, gluteofemoral fat percentage

Figure 2 shows the correlation between LAMSI and CL values. Among males, GFFP (*r*=−0.253, *p*=0.004) negatively correlated with CL values, while LASMI (*r*=−0.042, *p*=0.636) did not correlate with CL values. In contrast, among females, LAMSI (*r*=−0.205, *p*=0.037) negatively correlated with CL values, while GFFP (*r*=0.022, *p*=0.827) did not correlate with CL values.

## Discussion

In the present study, we systematically investigated the effects of muscle and fat mass in the thighs on brain Aβ deposition between males and females in a large cohort. We found that higher muscle mass, as reflected by the LASMI, was associated with a lesser risk of Aβ positivity in females. Higher fat mass, as reflected by the GFFP, was associated with a lesser risk of Aβ positivity in males. Thus, our findings suggest that there may be gender differences in the effects of body composition on Aβ deposition. Furthermore, our results may help in designing strategies for controlling body composition to prevent Aβ deposition.

Our first major finding was that higher muscle mass in the thighs was associated with a lesser risk of Aβ positivity in females. Previous studies have shown that sarcopenia is associated with incident probable AD dementia and cognitive impairment in older adults [35, 36]. However, information on the association between sarcopenia and AD biomarkers is lacking. Several possible mechanisms have been proposed to explain the relationship between muscle mass and brain Aβ positivity. Specifically, this relationship may be explained by the fact that Aβ-mediated neurodegeneration in the hypothalamus is involved in regulating dietary intake and energy expenditure [37], and systemic pro-inflammatory changes [38, 39]. However, we did not observe this relationship in males. To our knowledge, our findings are the first to report the gender-specific deleterious effects of sarcopenia on Aβ deposition. One study showed that sarcopenia only increased all-cause mortality in females [40]. It is important to identify the reason why females are more vulnerable to sarcopenia. Hormonal and socio-behavioral effects may contribute to gender differences. In particular, testosterone and estrogen may play crucial roles. Testosterone possesses anabolic properties, and estrogen modulates and prevents inflammation [41]. A decrease in testosterone level in elderly males and estrogen deficiency in elderly females triggers the discontinuance of anabolic reactions in males and increases inflammatory reactions in females. These characteristics are known to be distinct precipitating factors for sarcopenia in relation to gender [41]. In fact, the Framingham Heart Study found that higher levels of inflammatory proteins, such as interleukin, were associated with sarcopenia in females [42]. Hence, it was expected that sarcopenia in females would be closely associated with inflammation, which might lead to increased Aβ deposition.

Our second major finding was that higher fat mass in the thighs was associated with a lesser risk of Aβ positivity in males. The association of fat mass with Aβ might be explained by the distinct effects of fat on Aβ positivity depending on the fat tissue location. A previous study suggested that males have more abdominal fat than females, whereas females have more fat mass in their thighs than males [14]. Unlike abdominal fat, fat mass in the thighs is known to have beneficial effects on metabolic health, which is closely related to a higher level of adiponectin known as a fat tissue-specific hormone [15]. In fact, it has been reported that there are lower levels of adiponectin in males than in age-and BMI-matched females [43]. Growing evidence indicates that adiponectin possesses anti-inflammatory and insulin-sensitizing properties [15] and it has a protective role in the development of neurodegenerative diseases [44, 45]. In particular, a recent study revealed that higher levels of adiponectin were associated with higher Aβ levels in the cerebrospinal fluid, which suggested possible neuroprotective effects of adiponectin against Aβ positivity [46]. Thus, it was expected that a reduction in adiponectin levels might be a possible explanation for male-specific vulnerability following a reduction of fat mass in the thighs.

## Limitations

The strength of the present study is that we systematically investigated the effects of body composition on Aβ positivity with measurements of muscle and fat mass in the thighs in a large-sized cohort. However, our study has several limitations that should be addressed. First, since this was a cross-sectional study, it was difficult to determine the temporal relationships between the changes in body composition and Aβ deposition. Second, we were not able to assess repeated measurements over time, and then we did not cover the change or variability of muscle and fat mass. Third, we lacked more precise data of thigh muscle and fat mass, because we used LASMI and GFFP as proxy measures for muscle and fat mass in the thighs. However, existing findings show that GFFP highly correlates with actual values of thigh fat mass measured using DXA scans of the whole body [47], and LAMSI correlates with thigh muscle volume measured using MRI scans of the thigh [34]. Fourth, we used muscle and fat mass measured within 3 years before or after the Aβ PET. However, this limitation was alleviated to some extent by previous findings that the annual increase in Aβ is very low [48]. Finally, our participants were recruited from a population undergoing a comprehensive preventive health exam that was not covered by national medical insurance. The cohort was also restricted to participants undergoing DXA testing, which might have differed from the cohort of patients who did not undergo such testing. This study may have thus resulted in the enrollment of a healthier or more “health-seeking” population, which may also limit the generalizability of this study to other populations.

## Conclusions

In the present study, we highlight the importance of recognizing that gender differences may exist with respect to how specific body compositions may potentially protect against Aβ deposition. Furthermore, our findings suggest that the detection of changes in thigh muscle and fat mass in relation to gender are needed for early diagnosis and prevention of AD.

## Supplementary Information

**Additional file 1: Table S1.** Demographic variables of study participants stratified by cognitive stage.

## Data Availability

Anonymized data for our analyses presented in this report are available upon request from the corresponding authors.
