# Supplementary material for: Gender-specific relationship between thigh muscle and fat mass and brain amyloid-β positivity
Source: Alzheimers Res Ther. 2022 Oct 4;14:145. doi: 10.1186/s13195-022-01086-5 (PMC9531420; doi:10.1186/s13195-022-01086-5)
Supplement: Supplementary file 1 — Additional file 1: Table S1. Demographic variables of study participants stratified by cognitive stage. [file 13195_2022_1086_MOESM1_ESM.docx]

**Table S1.** Demographic variables of study participants stratified by cognitive stage

| Variables | | Males | | Females | |
| --- | --- | --- | --- | --- | --- |
|  | NC  (n = 62) | | MCI  (n = 72) | NC  (n = 61) | MCI  (n = 45) |
| *Demographics* |  | |  |  |  |
| Age, years | 70.9±7.4 | | 71.6±6.2 | 69.4±7.9 | 70.5±8.4 |
| Education, years | 12.4±4.5 | | 11.4±5.2 | 14.1±3.3 | 13.4±4.5 |
| *APOE*, *e4* carrier | 21 (33.9%) | | 26 (36.1%) | 15 (24.6%) | 18 (40.0%) |
| Hypertension | 35 (56.5%) | | 29 (40.3%) | 27 (44.3%) | 22 (48.9%) |
| Diabetes | 18 (29.0%) | | 14 (19.4%) | 6 (9.8%) | 11 (24.4%) |
| *Body composition* |  | |  |  |  |
| BMI, kg/m^2^ | 24.57±2.43^∗^ | | 23.51±2.73^∗^ | 23.73±2.91 | 23.18±2.98 |
| LASMI, kg/m^2^ | 5.67±0.49^∗^ | | 5.49±0.44^∗^ | 4.68±0.44 | 4.58±0.43 |
| GFFP, % | 18.67±3.52 | | 18.76±4.47 | 25.69±4.20 | 24.52±4.93 |
| *Aβ deposition* |  | |  |  |  |
| Aβ positivity | 13 (21.0%)^∗^ | | 31 (43.1%)^∗^ | 18 (29.5%)^∗^ | 23 (51.1%)^∗^ |
| Centiloid | 13.8±27.5^∗^ | | 38.7±48.8^∗^ | 17.7±28.8^∗^ | 41.2±42.6^∗^ |

Values are presented as mean ± standard deviation.

Abbreviations: Aβ (-), amyloid negative; Aβ (+), amyloid positive; n, number of patients whose data were available for analysis; BMI, body mass index; GFFP, gluteofemoral fat percentage; LASMI, lower extremity appendicular skeletal muscle mass index; NC, normal cognition; MCI, mild cognitive impairment.

^∗^Significant difference at *p*<0.05 between NC and MCI in the same gender.
